# 199. Polymorphisms in Key Regulatory Regions of the *bla* operon Correlate with the Cefazolin Inoculum Effect in Methicillin-Susceptible *Staphylococcus aureus* (MSSA)

**DOI:** 10.1093/ofid/ofab466.199

**Published:** 2021-12-04

**Authors:** Sara I Gomez-Villegas, Rafael Rios, Lorena Diaz, Lorena Diaz, An Q Dinh, Diana Panesso, Sandra Rincon, Lina P Carvajal, Maria I Reyes, Barbara E Murray, William R Miller, William R Miller, Kavindra V Singh, Jinnethe Reyes, Cesar A Arias

**Affiliations:** 1 University of Texas Health Sciences Center at houston, Houston, TX; 2 Universidad El Bosque, Bogota, Distrito Capital de Bogota, Colombia; 3 Center for Antimicrobial Resistance and Microbial Genomics, UTHealth, Houston, TX, Houston, Texas; 4 McGovern Medical School, Houston, TX; 5 Universidad el Bosque, Bogota, Distrito Capital de Bogota, Colombia; 6 Molecular Genetics and Antimicrobial Resistance Unit and International Center for Microbial Genomics, Universidad El Bosque, Bogota, Colombia, Bogota, Distrito Capital de Bogota, Colombia; 7 UTHSC at Houston Mc Govern Medical School, Houston, Texas; 8 Center for Antimicrobial Resistance and Microbial Genomics, UTHealth, Houston, TX; 9 CARMiG, UTHealth and Center for Infectious Diseases, UTHealth School of Public Health, HOU, TX; Molecular Genetics and Antimicrobial Resistance Unit and International Center for Microbial Genomics, Universidad El Bosque, BOG, COL, Houston, Texas

## Abstract

**Background:**

The cefazolin inoculum effect (CzIE), defined as Cz minimum inhibitory concentration ≥ 16 µg/ml at high inoculum (HI-MIC), has been associated with poor clinical outcomes in patients with MSSA bacteremia or osteomyelitis. The CzIE is correlated with the presence of the *blaZ* gene, one of the components of the *bla* operon encoding the BlaZ β-lactamase (type A, B, C or D). Other portions of the *bla* operon include *blaR* and *blaI* (encoding the antibiotic sensor and transcriptional repressor, respectively) and the intergenic region with operator and promoter sequences (**Figure 1**). In BlaR, residue 293 mediates signal transduction, and the Z and R dyads in the intergenic region are the DNA-binding sites for BlaI (**Figure 2**). Previous experiments have shown that the regulatory portions of the *bla* operon play a key role in the CzIE. Here, we investigated the association between the CzIE and specific variations in the regulatory sequences of the *bla* operon.

Figure 1. Functioning of the bla operon and the production of the staphylococcal β-lactamase BlaZ.

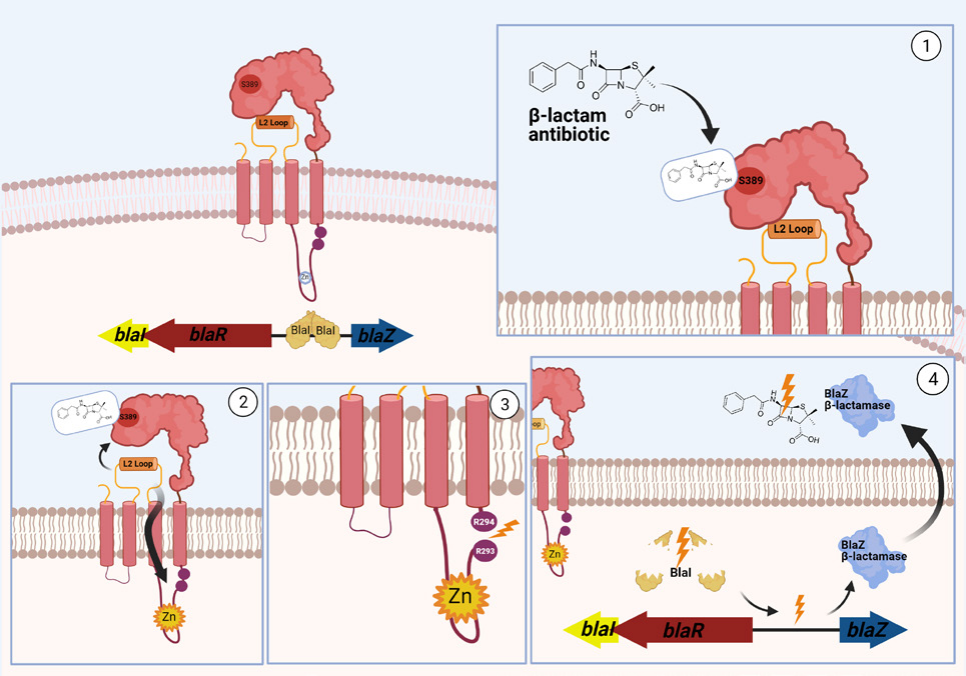

Figure 2. Structure and key regions of the intergenic region of the bla operon, incluiding the promoter and the BlaI DNA-binding regions (Z and R dyads).

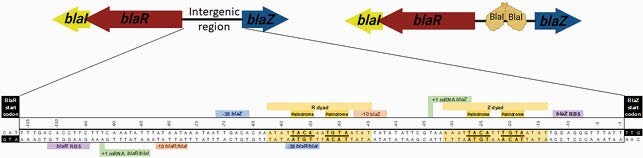

**Methods:**

A total of 437 MSSA containing *blaZ* were evaluated for the CzIE using broth microdilution at high inoculum. Using whole genome sequencing, the sequences of the *bla* operons were classified into cassettes based on unique changes in predicted amino acid sequences of BlaZ, BlaR and BlaI paired with specific nucleotide alterations in the intergenic region. The *bla* operon sequence of *S. aureus* ATCC29213 was used as reference (cassette 0).

**Results:**

Among 437 MSSA isolates, 46% exhibited the CzIE. We identified 55 unique *bla* cassettes. The *bla* cassettes were phylogenetically grouped in 7 clusters (**Figure 3**) which grouped cassettes with different BlaZ types and variations in the Z dyad, the -35 box, residue 293 of BlaR, and the *blaI* ribosomal binding site. Each cluster had an association to the CzIE and distinct Cz HI-MICs. The combination of: a BlaZ type A, C or D, an adenine in the position -66 of *blaZ* (-35 box of *blaZ*), a cytosine in the position -22 of *blaZ*(Z dyad), and either an arginine or a serine in position 293 of BlaR was a very strong predictor of the CzIE (**Figure 4**).

Figure 3. Phylogenetical organization of bla operon cassettes into clusters, their association with polymorphisms in key regulatory regions and the CzIE. GM Cz-MIC: Geometric mean of the Cefazolin MIC at high Inoculum.

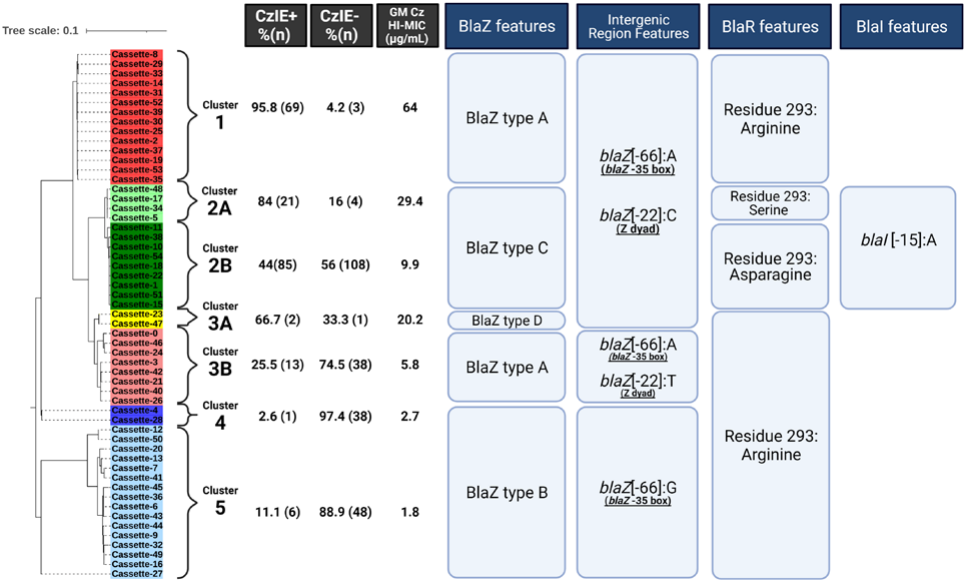

Figure 4. Variations of the bla operon, their association with the CzIE, their GM (Geometric Mean) of the Cefazolin MIC and the MIC distribution of the strains with each specific combination of polymorphisms.

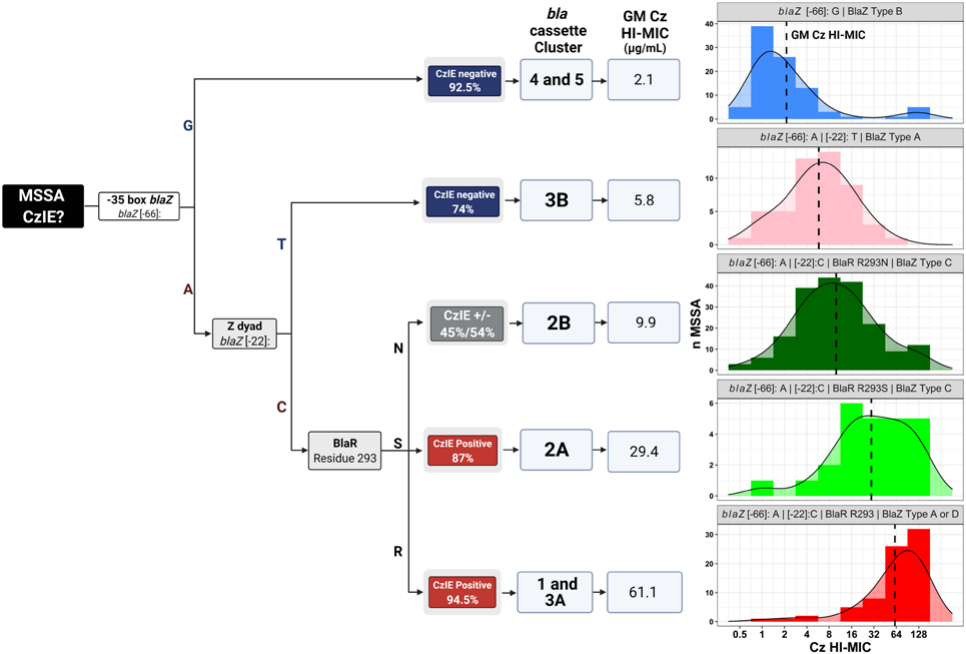

**Conclusion:**

Specific variations in regulatory portions of the *bla* operon, which are likely to influence BlaZ expression, are highly associated with the CzIE, supporting the notion that regulation of *blaZ* is the key factor responsible for the CzIE in MSSA.

**Disclosures:**

**Lorena Diaz, PhD** , Nothing to disclose **William R. Miller, MD** , **Entasis Therapeutics** (Scientific Research Study Investigator)**Merck** (Grant/Research Support) **William R. Miller, MD** , Entasis (Individual(s) Involved: Self): Scientific Research Study Investigator; Merck (Individual(s) Involved: Self): Grant/Research Support **Cesar A. Arias, M.D., MSc, Ph.D., FIDSA**, **Entasis Therapeutics** (Grant/Research Support)**MeMed Diagnostics** (Grant/Research Support)**Merk** (Grant/Research Support)

